# Implementation of GAN-Based, Synthetic T2-Weighted Fat Saturated Images in the Routine Radiological Workflow Improves Spinal Pathology Detection

**DOI:** 10.3390/diagnostics13050974

**Published:** 2023-03-03

**Authors:** Sarah Schlaeger, Katharina Drummer, Malek El Husseini, Florian Kofler, Nico Sollmann, Severin Schramm, Claus Zimmer, Jan S. Kirschke, Benedikt Wiestler

**Affiliations:** 1 Department of Diagnostic and Interventional Neuroradiology, School of Medicine, Klinikum Rechts der Isar, Technical University of Munich, Ismaninger Str. 22, 81675 Munich, Germany; 2 Department of Informatics, Technical University of Munich, Boltzmannstr. 3, 85748 Garching, Germany; 3 TranslaTUM—Central Institute for Translational Cancer Research, Technical University of Munich, Einsteinstr. 25, 81675 Munich, Germany; 4 Helmholtz AI, Helmholtz Zentrum München, Ingostaedter Landstrasse 1, 85764 Oberschleissheim, Germany; 5 TUM-NeuroImaging Center, Klinikum Rechts der Isar, Technical University of Munich, 81675 Munich, Germany; 6 Department of Diagnostic and Interventional Radiology, University Hospital Ulm, Albert-Einstein-Allee 23, 89081 Ulm, Germany

**Keywords:** magnetic resonance imaging, spine, generative adversarial network, T2-w fat saturated images, data augmentation

## Abstract

(1) Background and Purpose: In magnetic resonance imaging (MRI) of the spine, T2-weighted (T2-w) fat-saturated (fs) images improve the diagnostic assessment of pathologies. However, in the daily clinical setting, additional T2-w fs images are frequently missing due to time constraints or motion artifacts. Generative adversarial networks (GANs) can generate synthetic T2-w fs images in a clinically feasible time. Therefore, by simulating the radiological workflow with a heterogenous dataset, this study’s purpose was to evaluate the diagnostic value of additional synthetic, GAN-based T2-w fs images in the clinical routine. (2) Methods: 174 patients with MRI of the spine were retrospectively identified. A GAN was trained to synthesize T2-w fs images from T1-w, and non-fs T2-w images of 73 patients scanned in our institution. Subsequently, the GAN was used to create synthetic T2-w fs images for the previously unseen 101 patients from multiple institutions. In this test dataset, the additional diagnostic value of synthetic T2-w fs images was assessed in six pathologies by two neuroradiologists. Pathologies were first graded on T1-w and non-fs T2-w images only, then synthetic T2-w fs images were added, and pathologies were graded again. Evaluation of the additional diagnostic value of the synthetic protocol was performed by calculation of Cohen’s ĸ and accuracy in comparison to a ground truth (GT) grading based on real T2-w fs images, pre- or follow-up scans, other imaging modalities, and clinical information. (3) Results: The addition of the synthetic T2-w fs to the imaging protocol led to a more precise grading of abnormalities than when grading was based on T1-w and non-fs T2-w images only (mean ĸ GT versus synthetic protocol = 0.65; mean ĸ GT versus T1/T2 = 0.56; *p* = 0.043). (4) Conclusions: The implementation of synthetic T2-w fs images in the radiological workflow significantly improves the overall assessment of spine pathologies. Thereby, high-quality, synthetic T2-w fs images can be virtually generated by a GAN from heterogeneous, multicenter T1-w and non-fs T2-w contrasts in a clinically feasible time, which underlines the reproducibility and generalizability of our approach.

## 1. Introduction

Fat suppression to eliminate the signal from adipose tissue is frequently used in routine magnetic resonance imaging (MRI) [1]. The main advantages of fat-saturated (fs) images are the reduction of chemical shift artifacts and an improved tissue characterization by enhancing the fluid contrast [1,2]. Various different fat suppression or separation techniques exist, exploiting the different behavior of lipid protons and hydrogen protons from water during an MRI acquisition [1]. Common techniques use inversion recovery pulses (e.g., short tau inversion recovery (STIR), turbo inversion recovery magnitude (TIRM), spectral adiabatic inversion recovery (SPAIR)) or chemical shift encoding-based water-fat MRI (Dixon technique) [1,2,3].

The lack of radiation, its high soft tissue contrast, and the possibility for multiparametric, multiplanar, and three-dimensional imaging have made MRI an important imaging modality for the assessment of spinal pathologies, and its potential as a prognostic marker for different pathologies has been shown [4,5]. In spine MRI, T2-weighted (T2-w) sequences combined with fat saturation techniques enhance e.g., the assessment of bone marrow edema, inflammatory changes affecting vertebrae, intervertebral discs, and the cord, or paravertebral tissue abnormalities due to inflammation, trauma, or after surgery [6,7,8,9,10,11,12,13,14]. Thus, particularly for the assessment of acute spinal pathologies, T2-w fs images have become indispensable in the clinical routine [15]. Moreover, for the decision of whether a contrast agent is needed or not, T2-w fs are frequently pivotal [16]. Consequently, the additional acquisition of T2-w fs images next to conventional T1-w and non-fs T2-w images improves diagnostic performance in numerous spinal pathologies.

Nonetheless, in the clinical routine, T2-w fs sequences are frequently missing, e.g., due to time constraints or motion artifacts. Often, only the subsequent precise analysis of the acquired sequences, when the patient has already quit the scanner, raises the radiologist’s need for further T2-fs images to perform a more accurate diagnosis.

Recently, deep learning (DL) techniques have been emerging to augment existing medical imaging data. In particular, generative adversarial networks (GANs) can be used to virtually generate synthetic contrasts from various MRI datasets as input in a clinically feasible time [17,18,19,20,21,22,23,24]. For example, in work by Conte et al., a GAN was used to synthesize missing T1 and FLAIR brain MR images as input to a DL tumor segmentation model [25]. Moreover, dedicated fs sequences have already been synthesized using GANs [15,26,27,28].

In general, to bridge the gap between research and clinical implementation, there is a high need to evaluate these DL models on heterogenous, multicenter data by focusing on their reproducibility, generalizability, and diagnostic value in the real-world clinical setting [29,30]. Whereas the value of a physically acquired T2-w fs sequence for the improvement of pathology assessment at the spine is generally accepted [13], an evaluation of the diagnostic use of GAN-based, synthetic T2-w fs images in the routine radiological workflow is still missing.

Therefore, by simulating the clinical practice with a heterogenous dataset, we investigated the diagnostic value of an additional synthetic T2-w fs sequence based on a GAN for pathology assessment of the spine. We hypothesized that the synthetic T2-w fs sequence would help the radiologists to more accurately characterize various spinal pathologies compared to assessment merely based on T1-w and non-fs T2-w images. Hence, the additional diagnostic value of the synthetic protocol (T1-w, non-fs T2-w, and T2-w fs images) compared to assessment based on T1-w and non-fs T2-w images only was analyzed.

## 2. Materials and Methods

### 2.1. Synthesis of Sagittal T2-w fs Images

A GAN was trained to synthesize T2-w fs images from T1-w and non-fs T2-w images. The framework is based on the pix2pix architecture by Isola et al. [31].

In the following, the image generation process is described in more detail:

T1-w, T2-w, and T2-w fs images were linearly resampled to 1 × 1 mm in-plain resolution and rigidly co-registered using ANTs. The image intensities were capped at the 1st and 99th percentile and scaled to a [−1; 1] range. For the generator, we opted for a standard U-Net encoder-decoder architecture [32], with the addition of dropout layers in the decoder part to simulate noise, and the discriminator is patch-based [33]. While the discriminator D learns to differentiate between real and synthetic T2-w fs images (conditional on the input images) and is therefore driven by a binary cross-entropy (BCE) loss, the generator G is optimizing a joint loss:

Loss_G_ = (1−SSIM(real,synthetic)) + (λ × BCE(1,D(synthetic))) (1)

Here, SSIM is the structural similarity index measure, a metric capturing the similarity between two images [34]. A particular advantage of SSIM over standard metrics such as the L1 norm is its tolerance against imperfect registrations (as it is not pixel-based). In order to enforce the generator to create “realistic” images, the loss also includes the discriminator’s “judgment” on the synthetic image. λ is a hyperparameter balancing the two loss components and was empirically set to 50 in our study.

The training was conducted slice-wise (sagittal slices), with spatial (flipping, rotation) and intensity (gaussian smoothing, random noise) augmentations. As is standard, the discriminator and the generator were trained in turns, using the Adam optimizer with a learning rate of 2 × 10^-4^. The training was run for 25 epochs, where one epoch represented one loop over all training slices (in random order for each epoch).

Virtual generation of one T2-w fs dataset takes, on average, less than 5 min depending on the computing power. Most of this time is needed for image registration; the image synthesis by the GAN takes less than 30 s.

To allow for re-testing reproducibility and generalizability of our approach, the GAN model and one test case can be found in the following repository: https://doi.org/10.6084/m9.figshare.16627576

### 2.2. MRI Data for Generation of Synthetic T2-w fs Images

#### 2.2.1. Patient Population

There were 201 patients with MRI of the spine who were retrospectively identified. The local ethics commission approved the study design. Informed consent was waived due to the retrospective character.

#### 2.2.2. Training Data

Training data were retrospectively retrieved from 160 sagittal T1-w turbo spin echo (TSE), non-fs T2-w TSE, and T2-w TSE fs spine images of 96 patients. 31 datasets of 23 patients were excluded due to metal artifacts or poor image quality (only in the training data). The remaining 129 datasets from 73 patients originated from two in-house 3 T scanners (Ingenia and Achieva dStream, Philips Healthcare, Best, The Netherlands) with a similar protocol. Sequence parameters are given in Table A1.

#### 2.2.3. Testing Data

105 MRI datasets of 105 patients consisting of sagittal T1-w TSE and non-fs T2-w TSE scans were retrospectively identified in the institutional PACS starting with date 2020/10/01 and going backward by including all subsequent spine scans. Thereby, in-house scans and scans from other institutions (n = 50) were included. Four datasets were excluded due to missing data or data processing errors during export. Of note, significant artifacts e.g., due to foreign material or poor image quality, did not represent an exclusion criterion to assess the performance of the GAN even in these critical situations. The remaining 101 datasets originated from n = 38 scanners from three vendors (Philips Healthcare, Best, The Netherlands; Siemens Healthineers, Erlangen, Germany; GE Healthcare, Chicago, IL, USA). n = 41 datasets were acquired at 1.5 T, n = 60 datasets at 3 T. Mean/range of sequence parameters are given in Table A1.

### 2.3. Evaluation of the Diagnostic Value of Synthetic T2-w fs Images in the Radiological Workflow

#### 2.3.1. Radiological Readings

The 101 test datasets (T1-w, non-fs T2-w, and synthetic T2-w fs images) were investigated by two neuroradiologists (reader 1 with six years of experience, reader 2 with three years of experience). Prior to grading, in every dataset, a field of interest spanning five consecutive vertebral segments was defined, including cervical, thoracic, and lumbar spine segments. The diagnostic value of additional synthetic T2-w fs images was assessed by grading six different pathologies in the field of interest: bone marrow abnormalities (n = 61), spondylodiscitis expansion (n = 5), inflammatory Modic changes (n = 28), vertebral fractures (n = 21), spinal cord lesions (n = 15), and paravertebral tissue abnormalities (n = 25). Grading scores are given in Table 1. In order to simulate the radiological workflow as realistically as possible, the neuroradiologists first graded the pathologies on T1-w and non-fs T2-w images only. Then they added the respective synthetic T2-w fs and graded the pathologies again, consequently now incorporating all the information from T1-w, non-fs T2-w, and T2-w fs images. The neuroradiologists were blinded to scores of each other and any other imaging or clinical data from the patients.

#### 2.3.2. Reference Standard Definition

To determine a ground truth (GT) grading, subsequently, the pathologies on the same 101 test datasets were graded again in a combined assessment of both neuroradiologists, now incorporating the information provided by the real T2-w fs, pre- or follow-up scans, other imaging modalities, and clinical information; thus, creating a consensus GT assessment used as reference.

### 2.4. Statistical Analysis

Statistical analysis was performed with SPSS (version 27.0, IBM SPSS Statistics for MacOS, IBM Corp., Armonk, NY, USA) and Microsoft Excel (2021). A *p*-value of 0.05 was set as the threshold for statistical significance.

Additional diagnostic information of the synthetic protocol versus T1-w and non-fs T2-w images only was assessed using Cohen’s Kappa (ĸ) coefficient as a statistic to assess the inter-method reliability of qualitative, categorial items. Cohen’s ĸ coefficients for the agreement between grading based on the synthetic protocol with GT versus grading based on T1-w and non-fs T2-w images only with GT were calculated. We used the following interpretation of Cohen’s ĸ values between 0 and 1: below 0.20, poor agreement; 0.21 to 0.40, fair agreement; 0.41 to 0.60, moderate agreement; 0.61 to 0.80, substantial agreement; and above 0.81, almost perfect agreement [35]. Statistically significant differences between Cohen’s ĸ coefficients were evaluated using the Wilcoxon signed-rank test. The accuracy of grading was calculated, and corresponding significance was evaluated using McNemar’s test.

## 3. Results

The addition of the synthetic T2-w fs images to the imaging protocol led to a significantly higher agreement with the GT grading compared to pathology grading based on T1-w and non-fs T2-w images only (Cohens ĸ coefficients significantly higher for synthetic protocol; *p* = 0.043) (Table 2).

Accuracy for grading based on the synthetic protocol (with additional synthetic T2-w fs) was higher than for grading based on T1-w and non-fs T2-w images only, except for spinal cord lesions (Table 3). In particular, accuracy was significantly higher for grading inflammatory Modic changes (*p* = 0.034) (Table 3).

**Figure 1:**
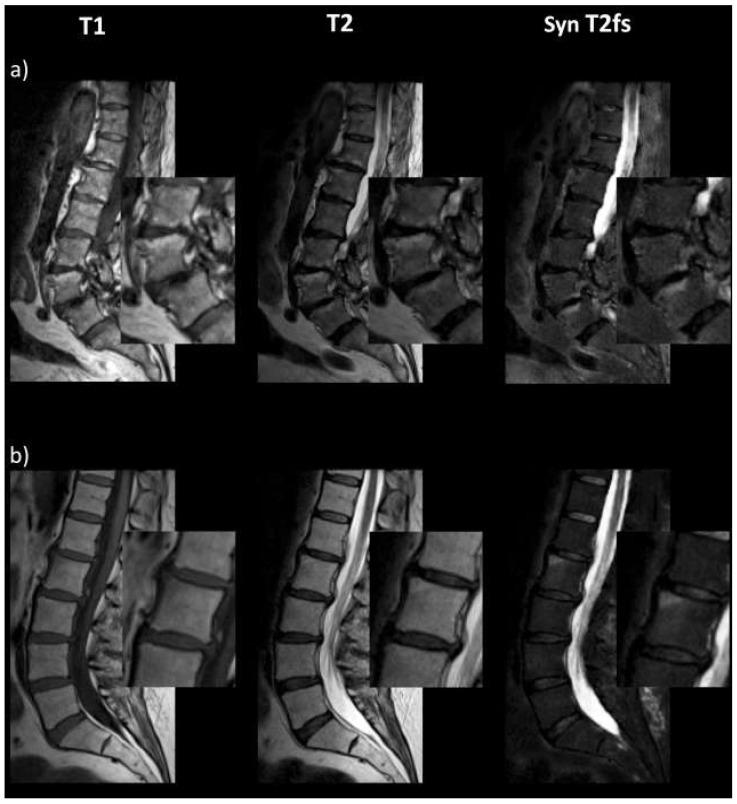
Synthetic T2-w fs images allow for better differentiation and characterization of inflammatory Modic changes (type 1) at the base plate of L4 and upper plate of L5 (**a**), as well as at the edge of upper plate L2 (**b**).

Figure 1 exemplarily shows the additional diagnostic value of the synthetic T2-w fs images for assessment of inflammatory Modic changes. In Figure 1a, the degenerative changes at the base plate of L4 and upper plate of L5 are clearly visible in the T2-w fs image. In contrast, the T2-w hyperintensities of the L3/4 endplates are easily distinguishable as fatty changes with the help of the additional synthetic T2-w fs image. Moreover, in Figure 1b, the subtle inflammatory Modic changes at the edge of the upper plate of L2 could likely be overseen when assessment is merely based on T1-w and non-fs T2-w images, whereas they are easily determinable in the T2-w fs image.

**Figure 2:**
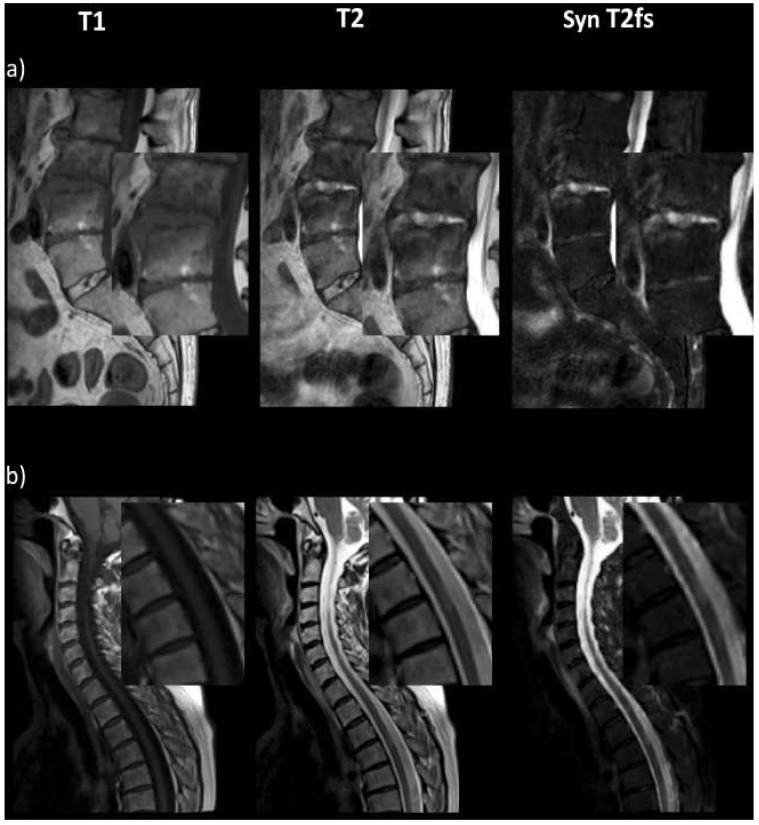
Synthetic T2-w fs images allow for better differentiation and characterization of spondylodiscitis and the associated fluid collection anterior of L4/5 (**a**), as well as cord lesions in the thoracic spine (**b**).

In Figure 2a, particularly the spondylodiscitis-associated fluid collection anterior of L4/5 only becomes obvious in the T2-w fs image. The same applies to the spinal cord lesions shown in Figure 2b.

## 4. Discussion

Our work demonstrates the value of GAN-based data augmentation in routine radiological spine assessment. Within clinically feasible time, we can generate high-quality, synthetic T2-w fs images from T1-w and non-fs T2-w contrasts. The addition of these virtual T2-w fs images to the imaging protocol adds significant diagnostic information. Hence, our work underlines the potential of a future implementation of GAN frameworks in the clinical setting by underlining the reproducibility, generalizability, and diagnostic value of our approach.

As the virtual generation of the respective synthetic T2-w fs images takes less than 5 min, the radiologist in charge has the possibility to virtually augment the available T1-w and non-fs T2-w data directly during image reading. By e.g., clicking on a graphical user interface (GUI) in the PACS system, consequently sending the input data to the GAN framework and receiving the synthetic T2-w fs contrast in less than 5 min, the possibility for a more accurate diagnosis of spine pathologies is given. Thereby, we could give access to the diagnostic benefit of T2-w fs images without the need for prolonged scan protocols or time-consuming rescanning of patients, decreasing the incidence of operator errors and patient motion artifacts. A retrospective generation of synthetic T2-w fs images allows for incorporation of the diagnostic benefit of fs images during late assessment, when initially a fs sequence has not been part of the protocol.

According to the evaluations of two independent neuroradiologists for all six pathologies, agreement with the GT was higher when using the synthetic protocol (with additional synthetic T2-w fs images) than when grading was based on T1-w and non-fs T2-w images only. Thus, the neuroradiologists could better detect and more accurately grade abnormalities after the addition of the synthetic T2-w fs images to the imaging protocol. These findings align with the general consensus regarding the value of an additional T2-w fs sequence in spine imaging. Particularly, for assessment of inflammatory Modic endplate changes (type 1), where MRI is generally known to provide a unique means to evaluate morphological changes of intervertebral discs and adjacent endplates [36], accuracy significantly increased when incorporating the synthetic T2-w fs sequences in the grading. Degenerative endplate changes might be subtle, and an additional T2-w fs significantly facilitates detection by replacing the time-consuming exact comparison between T1-w and non-fs T2-w images. In a review by Fields et al. [37], a lack of fat saturation in the MRI examination is seen as one cause for low and variable sensitivity of discography-concordant low back pain detection. Next to field strength, T2-w fs images improve the appearance of water and fat signal and, therefore, particularly facilitate the assessment of Modic type 1 endplate changes.

To ensure a wide range of clinical applications, the external validity of the employed GAN framework is of great importance. Previous work has already combined T1-w and non-fs T2-w images to virtually generate T2-w fs images using DL [15,26,28]. However, our study presents a GAN framework that is validated on a large and heterogenous multicenter dataset. As we trained our framework on images from only two in-house scanners and tested it on unseen, heterogenous data from 38 different MRI scanners, we could underline the generalizability of our approach.

The additional diagnostic value of synthetic images shown in this study could emphasize the use of GANs for data augmentation of spine imaging also in research settings. As formerly T2-w fs sequences were not routinely included in scan protocols of the spine, large datasets exist that lack the additional diagnostic value of fs images. For instance, the large epidemiological cohort study SHIP (“Study of Health in Pomerania”) only provides T1-w and non-fs T2-w images of the spine [38]. In this case, the retrospective generation of missing T2-w fs images opens diverse new research possibilities. The augmentation of a large spine dataset with fs images might foster a more accurate analysis of particularly acute pathologies such as bone marrow edema or vertebral fractures with regard to population-based research questions. Additionally, a common obstacle in computer vision, particularly in the medical field, is the lack of sufficiently diverse and large datasets for the training and testing of algorithms. This can lead to overfitting and a lack of generalizability of the employed models [39]. Recently, it was shown that GANs offer a novel approach for artificial data augmentation, e.g., eventually leading to more accurate classification of underrepresented classes in chest X-rays or improved generalizability of computed tomography (CT) segmentation tasks [40,41]. Moreover, in spine MRI, where data collection is expensive and scarce, network performance for segmentation tasks might potentially benefit from higher variability of the training data provided by GAN-based synthetic images.

It is known that synthetic images generated by neuronal networks, especially when a deconvolutional operation is included, might contain artifacts, such as checkerboard artifacts [42]. These checkerboard-like patterns in the image produced by the GAN itself might disturb the detection of subtle pathologies or might even mimic pathological changes. Therefore, particularly in medical imaging, it is crucial that images reflect reality. The high accuracy of grading based on the synthetic protocol might indicate the overall agreement with the reference standard, thus potentially underlining that no relevant errors in our synthetic images have influenced diagnostic performance.

The present study also has its limitations. First, the evaluations concentrated on six pathologies (bone marrow abnormalities, spondylodiscitis expansion, inflammatory Modic changes, vertebral fractures, spinal cord lesions, and paravertebral tissue abnormalities) for which it is known that sufficient fluid contrast is important for assessment. The generalizability for other pathologies still needs to be investigated. Second, the more accurate grading with additional synthetic T2-w fs images might also be in part explained by the fact that the corresponding sequence was added after the pathology had already been graded on T1-w and non-fs T2-w images, which means a second look for the image readers. However, the intention of the present study was to explicitly simulate the radiological workflow, in which the radiologist can decide whether she/he wants to make use of the additional diagnostic value of a synthetic T2-w fs and consequently initiates its generation by a GAN. Third, some interrater variability might be explained by the slightly different clinical experience of the two image readers.

## 5. Conclusions

To conclude, our work highlights the potential of GAN frameworks in routine radiological spine assessment. High-quality synthetic T2-w fs images can be virtually generated by a GAN from heterogeneous multicenter T1-w and non-fs T2-w contrasts in a clinically feasible time, which underlines the reproducibility and generalizability of our approach. The resulting virtual T2-w fs images significantly improve overall pathological assessment without the need for prolonged scan protocols or time-consuming rescanning.

## Figures and Tables

**Table 1:** Grading scores for the six different spine pathologies.

Pathologies	Grade				
	0	1	2	3	4
**Bone marrow abnormalities**	Absent	Focal	One-third of vertebral body	Two-thirds of vertebral body	Whole vertebral body or affection of pedicles/proc. spinosus
**Spondylodiscitis expansion**	Absent	One-third of vertebral body	Two-thirds of vertebral body	Whole vertebral body	-
**Juxtadiscal Modic changes (inflammatory; type 1)**	Absent	Present	-	-	-
**Vertebral fractures**	Absent	Acute (edema present)	Chronic	-	-
**Spinal cord lesions**	Absent	Present	-	-	-
**Paravertebral tissue abnormalities**	Absent	Inflammation	Hematoma	Other	-

**Table 2:** Inter-method agreement (Cohen’s Kappa (ĸ) coefficient; confidence interval (CI) of 95 %) ground truth (GT) with grading based on T1-w/non-fs T2-w images only and inter-method agreement GT with grading based on the synthetic protocol (T1-w, T2-w, and additional synthetic T2-w fs), respectively. ĸ coefficients were significantly higher for grading based on the synthetic protocol compared to grading based on T1-w and non-fs T2-w images only (*p* = 0.043).

Pathology	n (GT)	GT vs. T1-w/Non-fs T2-w [CI]	GT vs. Synthetic Protocol [CI]
Bone marrow abnormalities	61	**0.73** [0.67–0.78]	**0.74** [0.67–0.82]
Spondylodiscitis expansion	5	**0.35** [0.17–0.54]	**0.43** [0.14–0.72]
Juxtadiscal Modic changes (inflammatory; type 1)	28	**0.39** [0.29–0.49]	**0.68** [0.56–0.79]
Vertebral fracture	21	**0.77** [0.71–0.84]	**0.77** [0.68–0.87]
Spinal cord lesions	15	**0.47** [0.34–0.60]	**0.52** [0.34–0.69]
Paravertebral tissue abnormalities	25	**0.67** [0.59–0.75]	**0.73** [0.62–0.83]

**Table 3:** Accuracy in % of grading based on T1-w/non-fs T2-w and on the synthetic protocol (with additional synthetic T2-w fs). The accuracy of grading of Modic changes based on the synthetic protocol was significantly higher than grading based on T1-w and non-fs T2-w images only (*p* = 0.034) (*).

Pathology	n (GT)	Accuracy T1-w/Non-fs T2-w [%]	Accuracy Synthetic Protocol [%]
Bone marrow abnormalities	61	81.2	82.2
Spondylodiscitis expansion	5	94.6	95.0
Juxtadiscal Modic changes (inflammatory; type 1)	28	80.2 *	87.1 *
Vertebral fracture	21	91.6	92.1
Spinal cord lesions	15	90.1	90.0
Paravertebral tissue abnormalities	25	88.1	88.6

## Data Availability

The datasets used and/or analyzed during the current study are available from the corresponding author upon reasonable request.

## Appendix A

**Table A1:** Sequence parameters of GAN training dataset and testing dataset.

	Training Data 2 Internal Scanners			Test Data (Mean; Range) 5 Internal Scanners and 33 External Scanners	
	T1-w	non-fs T2-w	T2-w fs	T1-w	non-fs T2-w
**TR [ms]**	494	2517	2517	603; 13 −960	3176; 2400–6600
**TE [ms]**	8	100	100	11; 5–57	102; 80–126
**Slice gap [mm]**	3.3	3.3	3.3	3.4; 2.2–5.4	3.5; 2.2–5.4
**Averages**	1	1	1	1.5; 1–5	1.3; 1–3
**FOV xdim [mm]**	280	280	280	291; 48–420	291; 200–420
**FOV ydim [mm]**	280	280	280	293; 200–420	292; 200–420
**FOV zdim [mm]**	56	56	56	55; 30–256	55; 33–420
**Scan duration [s]**	154	186	186	155; 100–283	207; 102–349
